# Arthroscopic treatment of deep gluteal syndrome and the application value of high-frequency ultrasound

**DOI:** 10.1186/s12891-023-06863-3

**Published:** 2023-09-19

**Authors:** Guanjun Sun, Weili Fu, Qingshan Li, Yi Yin

**Affiliations:** 1 Department of Orthopedics, West China Hospital, Sichuan University, Sichuan Province, Chengdu, 610041 China; 2 Department of Joint Surgery, Suining Central Hospital, Sichuan Province, Suining City, 629000 China

**Keywords:** Deep gluteal syndrome, Arthroscopy, Release, Sciatic nerve

## Abstract

**Purpose:**

This study aimed to evaluate the efficacy of arthroscopic sciatic neurolysis for treating deep gluteal syndrome (DGS) and to analyse the application value of high-frequency ultrasound during perioperative period.

**Methods:**

Between June 2020 and February 2022, 30 patients with DGS who underwent failed conservative treatment were retrospectively analysed. Lateral arthroscopic exploration of the deep gluteal space and sciatic neurolysis were performed. In addition to pelvic X-ray, lumbar disc and hip magnetic resonance imaging (MRI), ultrasonography of the sciatic nerve was also performed in all patients. The visual analogue scale pain score (VAS), modified Harris hip score (mHHS) and Benson symptom-rating scale were used to evaluate the clinical efficacy. The correlation between preoperative sciatic nerve ultrasound and arthroscopic findings was analysed.

**Results:**

The median follow-up for these patients was 13 months (range,12–21 months). Preoperative ultrasonography showed precise morphological changes in 26 sciatic nerves of patients. The VAS score decreased from 5.0 (4.0, 6.0) preoperatively to 0.5 (0, 1.0) postoperatively (*p* < 0.001), and the mHHS increased from 64.0 (57.0, 67.0) preoperatively to 95.0 (93.0, 97.0) postoperatively (*p* < 0.001). The Benson symptom score was excellent in 15 cases, good in 12 cases, fair in 2 cases, poor in 1 case; thus, the score was excellent or good in 90% of the cases. Preoperative ultrasound diagnosis and intra-operative findings matched up in all cases. There were four cases of transient numbness in the posterior thigh.

**Conclusions:**

Arthroscopic sciatic neurolysis is a safe and effective treatment option for DGS patients who fail conservative treatment. Ultrasound diagnosis matched the arthroscopic findings perfectly. Preoperative Doppler ultrasound can assist surgical decision-making, guide intraoperative release.

## Introduction

Deep gluteal syndrome (DGS) is a condition in which the sciatic nerve is compressed in the deep gluteal space. It is characterized by nondiscogenic and extrapelvic entrapment of the sciatic nerve (SN), pain, paraesthesia, or radicular pain in the buttocks, hips, or rear thighs [1, 2]. Between the middle and deep fascia of the buttocks lies the deep gluteal space, which contains connective and adipose tissue. The borders of the deep gluteal space are as follows: the posterior border is the gluteus maximus, the anterior border is the trochanter and femoral neck, the medial border is the sacrotuberous ligament, and the lateral border is the femoral linea aspera. The upper border is the inferior margin of the ischial notch, and the lower border is the starting point of hamstring tendon [3].

DGS is difficult to diagnose, and there is currently no gold standard for diagnosis [4]. The diagnosis can only be established after other causes of hip or lower limb pain have been ruled out. Therefore, DGS is a ruled-out diagnosis. Previous studies have suggested a low incidence of DGS [5]. There is an estimated 6% to 17% prevalence of DGS in sciatica patients in the latest research [6, 7].

There may be a lack of uniform diagnostic criteria for DGS or a lack of familiarity among physicians. In addition to conventional pelvic X-ray, lumbar intervertebral disc and hip MRI, the use of high-frequency ultrasound examinations of nerves and muscles has gradually expanded [8]. Electromyography (EMG) is also used as one of the diagnostic tools in some studies [9]. There is also no gold standard treatment for DGS. There are three main types of treatment: medicine, rehabilitation, and operation. The procedure involves the open and arthroscopic release of the SN. Arthroscopic sciatic neurolysis is gaining popularity for its low risk of complications, minimal trauma, and rapid recovery [10, 11]. Arthroscopic surgery is still relatively understudied. There is still controversy regarding the indications for the operation, the standard operative procedure, the curative effect, and the safety of the procedure.

The purpose of this study was to (1) evaluate the efficacy and safety of arthroscopic sciatic neurolysis in patients with DGS who had failed to respond to conservative treatment; (2) explore the relationship between ultrasound diagnosis and intra-operative findings.

## Materials and methods

### General information

**Fig. 1:**
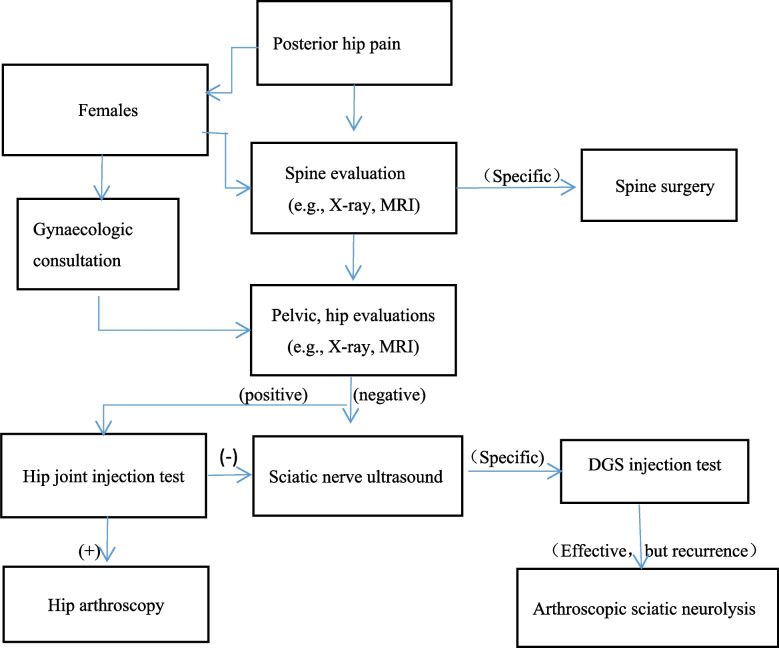
Diagnosis and treatment process protocol of DGS

The study involved 30 consecutive patients with DGS who failed conservative treatment between June 2020 and February 2022. The median age of the patients was 50 years (range, 29–77 years). There were 14 males and 16 females. Symptoms in these patients lasted for 7.5 months (range, 3–36 months). The indications for surgery included all patients who had been diagnosed with DGS. Another indication was that conservative treatment had been ineffective for three months. Nonsurgical treatment, including rest, activity modifications, oral nonsteroidal anti-inflammatories, muscle relaxants and physical therapy. The infiltration test around the tendon is valid for diagnosis and treatment in some people. This study was approved by the ethics committee of Suining Central Hospital. Our protocol is similar to that of Park et al. [12] (Fig. 1).

DGS can be diagnosed according to medical history and physical and auxiliary examinations. Clinical presentation and physical examination were performed as described by Martin et al. [13]. Clinical manifestations include 1) pain while walking, 2) pain while sitting (especially when sitting for > 30 min), 3) root pain in the lower back or buttocks, 4) paraesthesia, and 5) pain at night. Physical examinations included 1) tenderness on the sciatic notch, 2) the flexion-adduction-internal rotation test (FADIR) (positive when buttock pain was aggravated), 3) the Pace sign (pain and weakness with resisted abduction and external rotation of the hip), 4) the Laségue test (pain with straight leg raise testing to 90° hip flexion) and 5) the piriformis traction test in the sitting position. One or more symptoms and signs, along with an auxiliary examination, can lead to the diagnosis of DGS [13].

### Doppler ultrasound examination

A Canon colour Doppler ultrasound diagnostic instrument (Canon Aplio i900) was used with a probe frequency of 5–18 MHz. Patients were placed in the prone position and to completely expose their buttocks. The probe was obliquely inserted into the lateral upper quadrant of the buttocks. To mark ultrasonic surfaces, ilium (hyperechoic arc structure) was used. The probe was moved inwards and downwards in the longitudinal section until the greater sciatic notch was encountered. The probe was adjusted so that the long axis of the piriformis and the short axis of the sciatic nerve could be clearly and thoroughly visualized. The internal echo, the morphology and structure of the piriformis and SN, and their relationship were observed. This section was used to measure the thickness of the piriformis. A second adjustment was made so that the short axis of the piriformis and the long axis of the sciatic nerve could be displayed. In this section, the internal echo, boundary, and deformation of the SN were observed. The thickness of the sciatic nerve was measured at the lower edge of the piriformis muscle.

A comparison was made between the thickness of the piriformis and SN on the affected and healthy sides. Subsequently, the blood supply, morphology, and diameter of the SN were compared.

### Surgical procedure

General anaesthesia was administered in the lateral decubitus position. The greater trochanter and its apex and the SN were marked. The viewing portal (posterior portal) was located 3 cm posterior and 1 cm distal the apex of the trochanter. Another portal, designated the anterior portal, was located 5 cm superior of the apex of the trochanter on the femoral front line.

The two incisions were adjusted according to the shape of the patient. After marking and routine sterilization, a 0.5-cm incision was made at each marking position, the double valve tube was bluntly entered into the greater trochanter bursa, and the lens was placed in the bursa of the greater trochanter. The lens was then turned back to reveal the synovium and adipose tissue. Then, a trochar was introduced into the space through the anterior portal. The position and shape of SN were determined by blunt dissection under arthroscopic supervision. The fibrosis was removed by shaver and radiofrequency, and the SN was exposed.

The anatomical boundaries of the greater trochanter, piriformis, superior gemellus muscle, internal obturator, inferior gemellus muscle, and quadratus femoris were recognized. Branches of the inferior gluteal artery and the posterior femoral cutaneous nerve could be seen. The SN was again examined to determine whether there was any variation in the location and extent of nerve compression and to release the nerve based on the type of nerve compression. The epineurium was released if necessary, and the tendon insertion was partially cut. Flexion, extension, and internal and external rotation were performed to confirm that there was no mechanical or dynamic compression of the SN. Betamethasone and ropivacaine were injected around the incision. Surgical procedures differed from those described by Martin et al. [14] (Table 1).

**Table 1:** Key steps of arthroscopic release of the sciatic nerve

1. Enter the deep gluteal space
2. Identify and explore the sciatic nerve
3. Debridement of the deep gluteal space
4. Initial evaluation of the sciatic nerve
5. Identify and manage peripheral vessels of sciatic nerve
6. Identify the piriformis muscle, explore, and release the sciatic nerve here
7. Identify the short external rotation muscle group, explore, and release the sciatic nerve here
8. Identify the quadratus femoris, explore, and release the sciatic nerve
9. Explore the tension of the sciatic nerve, look for hidden compression and release them
10. Flexion, extension, internal and external rotation to determine the sciatic nerve without mechanical and dynamic compression

### Postoperative rehabilitation and assessment

After operation, the ankle pump and quadriceps femoris muscles were isometrically contracted to prevent deep vein thrombosis (DVT), and local ice compresses were applied. The patients were treated with nonsteroidal drugs also. Post-operatively, positions that put tension on the SN (e.g. combined knee extension and hip flexion) were avoided. Local massage was available three weeks later, and a standard physical therapy program was available six weeks later. All patients were required to see the doctor at 1 month, 3 months and 12 months after the operation and then saw a doctor by themselves according to the patient's condition. The shortest follow-up time was 12 months, and the longest was 21 months. The clinical assessment was performed using the VAS score and the mHHS [15]. Patient satisfaction was assessed using the Benson symptom rating scale [16]. There were four categories of outcomes: excellent, good, fair and poor.

### Statistical analysis

Data were entered into Excel and analysed with SPSS 21.0 software. α = 0.05 as the test level for comparison between groups. The Wilcoxon nonparametric rank sum test was used to describe the measurement data of nonnormal distribution, described by the median (25%, 75%). Categorical data are presented as the rate (%).

## Results

### Preoperative symptoms, signs, and results of Doppler ultrasound examination

**Fig. 2:**
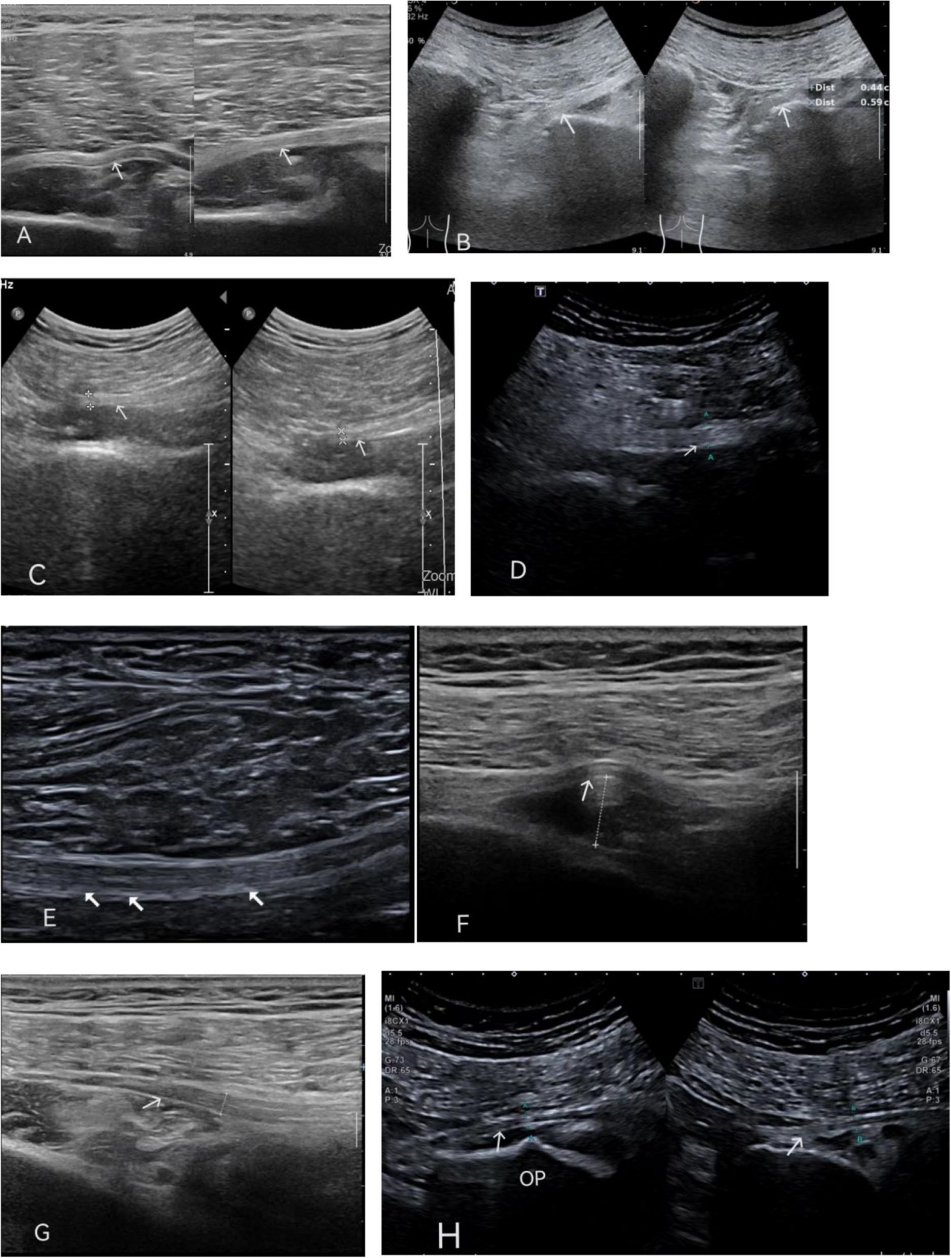
Preoperative ultrasound of various morphological changes in the sciatic nerve (SN) (the SN are marked with white arrows). **A** SN distorted with inhomogeneous border. **B** SN thickened (left). **C** SN thinned (right). **D** Gluteal muscle echo inhomogeneous with unclear SN border. **E** Thickness of SN bundles and not uniform. **F** Superior gemellus muscle oedema and SN thinning. **G** Synovial hyperplasia. **H** Osteophyte (OP)

The median follow-up was 13 months (range, 12–21 months). All patients were reported to have hip pain, and the preoperative symptoms and signs are shown in Table 2. Doppler ultrasound showed various pathological changes (Table 3 and Fig. 2).

**Table 2:** Preoperative symptoms and signs

Clinical presentations	Number
Preoperative symptoms	
Sit pain	25(83.3%)
Walking pain	24(80.0%)
Night pain	20(66.7%)
Root pain	4(13.3%)
Paraesthesia	5(16.7%)
Preoperative signs	
Sciatic notch tenderness	25(83.3%)
FADIR	11(36.7%)
Pace sign	17(56.7%)
Seated piriformis test	14(46.7%)
Laségue test	2(6.7%)

**Table 3:** Results of Doppler ultrasound

Preoperative ultrasound performance	Number
The piriformis muscle and sciatic nerve thickened	8
Only the sciatic nerve thickened	6
Sciatic nerve thinned	4
Sciatic nerve bundles not uniform in thickness	3
Superior gemellus muscle oedema and sciatic nerve thinned	2
Sciatic nerve distorted	2
Synovial hyperplasia	2
Sciatic cortex discontinuous and osteophyte formation	2
Gluteal muscle echo inhomogeneous with unclear Sciatic nerve border	1
Total	30

### Pain and function improved significantly after arthroscopic sciatic nerve release

The mean VAS scores decreased by 4.5 points (*p* < 0.001), and the mHHS increased by 31 points at 1 year follow-up (Table 4). Postoperative pain and function improved significantly. Benson's symptom-rating scale was excellent in 15 cases, good in 12 cases, fair in 2 cases, and poor in 1 case; thus, excellent or good scores were achieved in 90% of cases. There were four cases of transient posterior thigh numbness (Table 5). In the poor group (1 case), the echo of the gluteal muscles was inhomogeneous, and the boundary of the SN was not clear. In the fair group (2 cases), Doppler ultrasound showed that the SN bundle was not uniform in thickness. The postoperative efficacy was excellent in all other cases (ultrasound showed abnormal SN morphology or other hyperplasia).

**Table 4:** Comparison of VAS and mHHS pre- and postoperation

	VAS	mHHS
Preoperation	5.0(4.0,6.0)	64.0(57.0,67.0)
Postoperation	0.5(0,1.0)	95.0(93.0,97.0)
Z	-4.826	-4.784
P	< 0.001	< 0.001

A *P* value <0.05 was considered statistically significant

**Table 5:** Benson symptom-rating scale

Outcome	Symptoms	Number (%)
Excellent	No pain with prolonged periods of sitting (> 30 min), strenuous activity, bending, twisting, stairs, rapid walking, jogging	15 (50%)
Good	No pain with short periods of sitting (≤ 30 min) or daily activities or mild pain with prolonged periods of sitting or strenuous activity	12 (40%)
Fair	Occasional mild pain with short periods of sitting or normal daily activities or moderate pain with prolonged sitting or strenuous activity	2 (6.7%)
Poor	Severe pain with short periods of sitting or normal daily activities, little change from preoperative level of pain associated with sciatic nerve	1 (3.3%)

### Pathological changes in DGS under arthroscopy

**Fig. 3:**
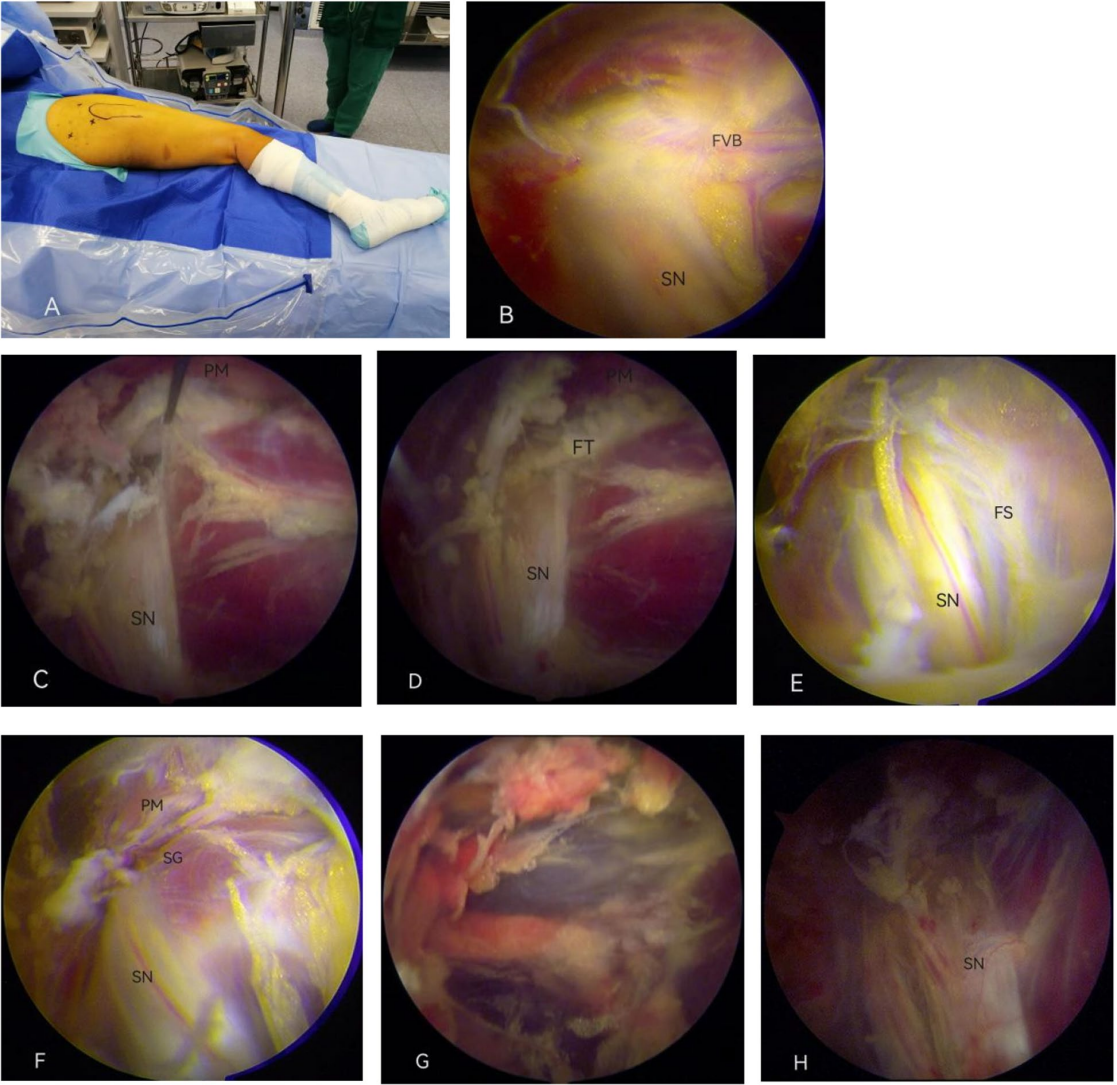
Intraoperative pathological changes. **A** Surgical position. **B** Fibrovascular bundle (FVB). **C** Piriformis muscle (PM). **D** Fibrous tissue (FT). **E** Fibrous scar (FS). **F** Superior gemellus muscle (SG). **G** Extensive synovial hyperplasia. **H** After synovial tissue debridement

The pathological changes in DGS during the operation are shown in Table 6. There were 15 cases of fibrous and vascular bundles, eight cases of piriformis hypertrophy, two cases of extensive synovial hyperplasia in the deep gluteal space, two cases of osteophyte formation, two cases of dynamic stimulation of the gemellus muscle-internal obturator complex, and one case without definitive findings (Fig. 3). Arthroscopic findings and ultrasound diagnosis matched up in all cases.

**Table 6:** Causes of sciatic nerve entrapment under arthroscopy

Compromising structure	Number
Fibrous and fibrovascular bands	15
Piriformis muscle hypertrophy	8
Synovial hyperplasia	2
Osteophyte formation	2
Dynamic stimulation of the gemellus muscle-internal obturator complex	2
No definite lesion	1
Total	30

### Complications

There were no infections, DVTs and sciatic nerve palsies in this group. But 4 cases had transient posterior femoral cutaneous nerve altered sensation.

## Discussion

DGS is a group of conditions in which the sciatic nerve is compressed by any structure in the deep gluteal space. The main symptoms include pain in the buttocks caused by pressure on the sciatic nerve and difficulty sitting for long periods. Pain is aggravated by prolonged walking and exercise of the hip. Radicular pain may occur on the affected side of the leg, similar to pain caused by lumbar disc disease [9]. Therefore, it is difficult to diagnose and treat since any structure in the deep gluteal space can cause mechanical or dynamic compression of the sciatic nerve. This study showed that arthroscopic sciatic neurolysis was a safe and effective treatment option for DGS patients who failed conservative treatment. Preoperative ultrasound could show a variety of sciatic neuropathies, which can be used as a routine examination for DGS and could assist surgical decision-making, localize intraoperative release.

Furthermore, there is no standard treatment. Currently, there are three main types of treatment: medicine, rehabilitation, and operation. The surgical procedure involves open or arthroscopic release of the sciatic nerve. Arthroscopic sciatic neurolysis is gaining popularity due to its low risk of complications, minimal trauma, and rapid recovery [10, 11]. Recent studies have reported 83–93% success rates with arthroscopic treatment of DGS [11, 17–19]. The excellent and good rates were 90% in our study, which was similar to the literature reports. In previous studies [11, 17–21], no or low complications have been reported, including infection, VTEs, nerve palsy, postoperative pain aggravation and recurrence, and intra-abdominal fluid extravasation (IAFE). None of these complications were observed in this series. But there were 4 patients (4/30, 13.3%) with posterior thigh numbness. Given the extent of the numbness, it could be femoral posterior cutaneous nerve injury [22]. However, they all recovered within 3 months after surgery. During arthroscopic surgery, we exposed the femoral posterior cutaneous nerve in some patients, the symptoms may be related to intra-operative exploration and stimulation.

The application of ultrasound in DGS is controversial. The 2012 Expert Consensus of the European Society for Musculoskeletal Radiology [23] concluded that musculoskeletal ultrasound was appropriate only to examine fluids, extra-articular snapping hips, synovitis, effusion, cysts, exercise-induced hernias, and severe muscle injuries. Musculoskeletal ultrasound was deemed not indicated for intra-articular snapping hip, osteoarthritis, and labral tears, also not indicated for low-grade muscle injuries, psoas tendon problems, trochanteric pain, sciatica and growing pain. Comparing the diagnostic value of MRI with that of Doppler ultrasonography in piriformis syndrome, Min et al. [24] concluded that both ultrasonography and MRI can clearly show the piriformis and sciatic nerve and that both qualitative and quantitative measurements can be used. Doppler ultrasound can aid in the clinical diagnosis of piriformis syndrome. Presently, the preferred examination for DGS is MRI, especially a 3 T MRI with a high resolution, which can reveal the deep gluteal vascular fibre band. Our clinical experience shows that conventional MRI scans can identify lesions in the hip joint, but they can sometimes miss lesions in the deep gluteal space. EMG and nerve conduction study are of doubtful value, which may demonstrate conduction abnormalities and denervation potentials,can’t provide more information for DGS. In some positions that cause SN compression, such as the FADIR test, electromyography may have some specificity and sensitivity [12, 24]. According to this study, Doppler ultrasound can be used as an effective supplement for DGS. The shape, thickness and involved part of the sciatic nerve can be determined using Doppler ultrasound. This can indicate that there could be tissue entrapment or stimulation in this part of the nerve, which is beneficial for targeted release during exploration during the operation. A more accurate curative effect was observed after the operation, and dynamic compression had a higher diagnostic advantage. Comparing arthroscopic findings and ultrasound diagnosis, the two matched up in all cases. So ultrasound can also be an essential reference for intraoperative release.

There are some differences in intraoperative release. Myung and Dong [11, 12] suggested that the piriformis muscle needs to be transected in addition to routine exploration for release. Other scholars [17, 25] have not specified whether to cut the tendon but have referred to the release of the tissues that produce the compression. To review the current literature and combine our clinical results after releasing the entire sciatic nerve of the deep gluteal space and to investigate whether there was dynamic entrapment in flexion-adduction-pronation and, if so, whether the tendon was partially or entirely severed until the compression was completely relieved. Partial transection of the piriformis or superior gemellus muscle tendon was performed in only two cases in our study. The sciatic nerve was distorted under the piriformis muscle in 18 patients, as shown by Doppler ultrasound before the operation. This study proved that the myolemma, not the muscle, was responsible for the compression. If release of the tendon is needed, special attention should be given to the extent to which the terminal branch of the medial circumflex femoral artery [26] enters the femoral head at the insertion of the internal obturator muscle, as it can easily lead to necrosis of the femoral head after injury.

## Strengths and limitations

Previous studies have not used ultrasound to guide surgery or assist surgical decision-making. This study analysed the preoperative ultrasound diagnosis and intra-operative findings the results demonstrated that the two matched up in all cases. Preoperative ultrasound can be a guide for intraoperative location and release.This study provides a new reference for the diagnosis and treatment of DGS. The following are some of the limitations of the study: 1) the number of cases was insufficient, which may lack of power; 2) Ultrasound is operator dependent, so the results of ultrasound may be somewhat subjective, which may have some effect on the result.

## Conclusion

Arthroscopic sciatic neurolysis is a safe and effective treatment option for DGS patients who fail conservative treatment. Ultrasound can be used as a routine examination for DGS. Doppler ultrasound before surgery can aid surgical decision-making, guide intraoperative release.

## Data Availability

The data and materials analysed during the current study are available from the corresponding author on reasonable request.

## Abbreviations

DGS: Deep gluteal syndrome
MRI: Magnetic resonance imaging
VAS: Visual analogue scale
mHHS: Modified Harris hip score
FADIR: Flexion-adduction-internal rotation
DVT: Deep vein thrombosis
EMG: Electromyography
IAFE: Intra-abdominal fluid extravasation
SN: Sciatic nerve
FVB: Fibrovascular bundle
PM: Piriformis muscle
FT: Fibrous tissue
FS: Fibrous scar
SG: Superior gemellus muscle
OP: Osteophyte

## References

[1] Martin HD, Palmer IJ, Hatem MA, Nho S, Leunig M, Larson C, Bedi A, Kelly B (2015). Deep gluteal space. Hip Arthroscopy and Hip Joint Preservation Surgery.

[2] Carro LP, Hernando MF, Cerezal L, Navarro IS, Fernandez AA, Castillo AO (2016). Deep gluteal space problems:piriformis syndrome, ischiofemoral impingement and sciatic nerve release. Muscles Ligaments Tendons J.

[3] Leite Maria J, Pinho André R, Silva Miguel R (2022). Deep gluteal space anatomy and its relationship with deep gluteal pain syndromes. Hip Int.

[4] Kay J, de Sa D, Morrison L (2017). Surgical management of deep gluteal syndrome causing sciatic nerve entrapment: a systematic review. Arthroscopy.

[5] Hopayian K, Heathcote J (2019). Deep gluteal syndrome: an overlooked cause of sciatica. Br J Gen Pract.

[6] Singh US, Meena RK, Singh CAK (2013). Prevalence of piriformis syndrome among the cases of low back/buttock pain with sciatica: a prospective study. J Med Soc.

[7] Kean Chen C, Nizar AJ (2013). Prevalence of piriformis syndrome in chronic low back pain patients. A clinical diagnosis with modified FAIR test. Pain Pract.

[8] Hernando MF, Cerezal L, Pérez-Carro L (2015). Deep gluteal syndrome: anatomy, imaging, and management of sciatic nerve entrapments in the subgluteal space. Skeletal Radiol.

[9] Kazuha, Soshi, Ajaykumar, et al. Deep gluteal syndrome is defned as a non‑discogenic sciatic nerve disorder with entrapment in the deep gluteal space: a systematic review. Knee Surg Sports Traumatol Arthrosc. 2020:3. 10.1007/s00167-020-05966-x. 10.1007/s00167-020-05966-x 32246173

[10] Yoon SJ, Park MS, Matsuda DK, Choi YH (2018). Endoscopic resection of acetabular screw tip to decompress sciatic nerve following total hip arthroplasty. BMC Musculoskelet Disord.

[11] Ham DH, Chung WC, Jung DU (2018). Effectiveness of endoscopic sciatic nerve decompression forthe treatment of deep gluteal syndrome. Hip Pelvis.

[12] Park MS, Yoon SJ, Jung SY (2016). Clinical results of endoscopic sciatic nerve decompression for deep gluteal syndrome: mean 2-year follow-up. BMC Musculoskelet Disord.

[13] Martin HD, Shears SA, Johnson JC, Smathers AM, Palmer IJ (2011). The endoscopic treatment of sciatic nerve entrapment/ deep gluteal syndrome. Arthroscopy.

[14] David Martin, Manoj Reddy, Hoyos. Deep gluteal syndrome. J Hip Preserv Surg. 2(2):99–107. 10.1093/jhps/hnv029. Mini Symposium. 10.1093/jhps/hnv029 PMC4718497 27011826

[15] Byrd JW, Jones KS (2000). Prospective analysis of hip arthroscopy with 2-year follow-up. Arthrosc J Arthrosc Relat Surg Off Publ Arthrosc Assoc N Am Int Arthrosc Assoc.

[16] Benson ER, Schutzer SF (1999). Posttraumatic piriformis syndrome: diagnosis and results of operative treatment. J Bone Joint Surg Am.

[17] Martin HD, Shears SA, Johnson JC, Smathers AM, Palmer IJ (2011). The endoscopic treatment of sciatic nerve entrapment/deep gluteal syndrome. Arthroscopy.

[18] Park MS, Jeong SY, Yoon SJ (2019). Endoscopic sciatic nerve decompression after fracture or reconstructive surgery of the acetabulum in comparison with endoscopic treatments in idiopathic deep gluteal syndrome. Clin J Sport Med.

[19] Ilizaliturri VM, Arriaga R, Villalobos FE, Suarez-Ahedo C (2018). Endoscopic release of the piriformis tendon and sciatic nerve exploration. J Hip Preserv Surg.

[20] Aguilera-Bohórquez B, Ramirez S, Cantor E (2020). Intra-abdominal fluid extravasation: is endoscopic deep gluteal space exploration a risk factor?. Orthop J Sports Med.

[21] Kocher MS, Frank JS, Nasreddine AY (2012). Intra-abdominal fluid extravasation during hip arthroscopy: a survey of the MAHORN group. Arthroscopy.

[22] Jiamjunyasiri A, Tsutsmi M, Muro S (2023). Origin, course, and distribution of the posterior femoral cutaneous nerve and the spatial relationship among its branches. Anat Sci Int.

[23] Klauser AS, Tagliafico A, Allen GM (2012). Clinical indications for musculoskeletal ultrasound:a Delphi-based consensus paper of the European Society of Musculoskeletal Radiology. Eur Radio.

[24] Ning M, Yao JC, Yang PJ (2021). Value of Ultrasound and MRI in Diagnosising Piriformis Syndrome. Chin J CT MRI.

[25] Metikala S, Sharma V (2022). Endoscopic Sciatic Neurolysis for Deep Gluteal Syndrome: A Systematic Review. Cureus.

[26] Gautier E, Ganz K, Krügel N (2000). Anatomy of the medial femoral circumflex artery and its surgical implications. J Bone Joint Surg Br.

